# Ran Involved in the Development and Reproduction Is a Potential Target for RNA-Interference-Based Pest Management in *Nilaparvata lugens*

**DOI:** 10.1371/journal.pone.0142142

**Published:** 2015-11-10

**Authors:** Kai-Long Li, Pin-Jun Wan, Wei-Xia Wang, Feng-Xiang Lai, Qiang Fu

**Affiliations:** State Key Laboratory of Rice Biology, China National Rice Research Institute, Hangzhou 310006, China; Kansas State University, UNITED STATES

## Abstract

Ran (RanGTPase) in insects participates in the 20-hydroxyecdysone signal transduction pathway in which downstream genes, *FTZ-F1*, *Krüppel-homolog 1 (Kr-h1)* and *vitellogenin*, are involved. A putative *Ran* gene (*NlRan*) was cloned from *Nilaparvata lugens*, a destructive phloem-feeding pest of rice. NlRan has the typical Ran primary structure features that are conserved in insects. *NlRan* showed higher mRNA abundance immediately after molting and peaked in newly emerged female adults. Among the examined tissues ovary had the highest transcript level, followed by fat body, midgut and integument, and legs. Three days after ds*NlRan* injection the NlRan mRNA abundance in the third-, fourth-, and fifth-instar nymphs was decreased by 94.3%, 98.4% and 97.0%, respectively. *NlFTZ-F1* expression levels in treated third- and fourth-instar nymphs were reduced by 89.3% and 23.8%, respectively. In contrast, *NlKr-h1* mRNA levels were up-regulated by 67.5 and 1.5 folds, respectively. *NlRan* knockdown significantly decreased the body weights, delayed development, and killed >85% of the nymphs at day seven. Two apparent phenotypic defects were observed: (1) Extended body form, and failed to molt; (2) The cuticle at the notum was split open but cannot completely shed off. The newly emerged female adults from ds*NlRan* injected fifth-instar nymphs showed lower levels of *NlRan* and *vitellogenin*, lower weight gain and honeydew excretion comparing with the blank control, and no offspring. Those results suggest that *NlRan* encodes a functional protein that was involved in development and reproduction. The study established proof of concept that *NlRan* could serve as a target for dsRNA-based pesticides for *N*. *lugens* control.

## Introduction

RanGTPase belongs to the Ras superfamily of small GTPases, and is essential for the translocation of RNA and proteins through the nuclear pore complex. It possesses a distinctive acidic C terminal D(E)E(D)DD(E)DL motif and predominantly localizes in the nucleus [1, 2]. Ran switches between the GDP-bound (RanGDP) and GTP bound (RanGTP) states for its activity. RanGDP combines with nuclear transport factor 2 and is transported into the nucleus [3, 4]. Once inside, RanGDP switches to RanGTP and is transported into the cytoplasm. Therefore, Ran plays a key role in controlling nucleocytoplasmic trafficking. However, nucleocytoplasmic transport is only one of Ran’s functions. Studies have indicated that it plays a wide range of roles in coordinating nuclear functions throughout the cell cycle, including nuclear assembly and DNA replication [5] and mitotic spindle assembly [6]. Ran was linked to cancer [7, 8] and was a potential therapeutic target for cancer cells [9]. It played a role in shrimp immunity against virus infection [10, 11]. Some recent researches demonstrated that Ran protein played an important role in plant development and mediated plant responses to the environment [12, 13]. In insects, Ran participated in the 20-hydroxyecdysone (20E) signal transduction pathway by regulating the location of ecdysone receptor-B1 (EcR-B1) in *Helicoverpa armigera*. RNAi knockdown of *Ha-Ran* resulted in suppression of other 20E regulated genes and blocked the nuclear location of EcR-B1 [14]. Although Ran has been well studied in *H*. *armigera*, the roles of Ran in hemiptera insect species have not been documented until now.

The brown planthopper, *Nilaparvata lugens* (Stål), is one of the most destructive insect pests of rice in Eastern and Southeastern Asia. The insects suck directly from the phloem tissue of growing rice plants causing wilting or drying of the crop, which is referred as “hopperburn” [15, 16]. Since 2005, rice planthoppers caused millions of hectares of rice to fail every year in major rice producing countries of Asia including Vietnam, Indonesia, China, South Korea and Japan [17]. Considerable quantities of insecticides are applied in attempts to control *N*. *lugens* each season with serious consequences of insecticide resistance development [18, 19]. To efficiently control *N*. *lugens* and successfully avoid insecticide resistance, novel control strategies must be explored.

RNA interference (RNAi) is a powerful tool for studying gene function through regulating gene expression at the mRNA level by double-stranded RNA (dsRNA) [20, 21], and potentially, for developing novel pest management strategies by plant-mediated RNAi [22–24]. Systemic RNAi *in vivo* has been successfully used to study a number of genes functioning in insects [25]. For *N*. *lugens* in particular, RNAi could be used in silencing vital genes by injection of dsRNA [26]. Suppression of chitin synthase 1 (CHS1) and CHS1a in *N*. *lugens* resulted in abnormal morphology and death [27]. Silencing of *NlEcR* expression by *in vivo* injection of dsRNA caused phenotypic defects in molting and resulted in high mortality (87–100%) of treated nymphs [28]. Knockdown of *Nlflightin* changed the indirect flight muscle and male dorsal longitudinal muscle structure [29]. *NlCPR*-dsRNA injection resulted in increased susceptibility of *N*. *lugens* to beta-cypermethrin and imidacloprid [30].

In the present study, we characterized the *Ran* gene in *N*. *lugens* and analyzed its temporal and spatial expression profiles. Furthermore, gene knockdown was performed through *NlRan*-dsRNA injection, and the insecticidal action was investigated. Since Ran participates in the 20E signal transduction pathway, in which the nuclear receptor FTZ-F1 is involved [14], and FTZ-F1 probably is also involved in juvenile hormone (JH) biosynthesis [31, 32]. The expression of *Krüppel-homolog 1* (*Kr-h1*) is responsive to JH and promotes vitellogenesis and oocyte maturation [31, 33, 34]. The effect of *NlRan* knockdown on the transcript levels of *NlFTZ-F1* and *NlKr-h1* also was examined. It was found that dsRNA injection significantly silenced the gene expression, delayed nymphal growth, affected nymphal molting and caused lethality. More importantly, the inhibition of ovary development by *Ran*-dsRNA injection resulted in no offspring.

## Materials and Methods

### Insect culture

*N*. *lugens* used in the study were stablished from a field collection in Fuyang (119.95E, 30.07N), Zhejiang, China, in 1996, and reared on rice (*Oryza sativa*) variety Taichung Native 1 (TN1, an insect susceptible rice variety) in China National Rice Research Institute under controlled conditions of 28±1°C, 80±10% relative humidity and a 16/8 h light/dark photoperiod for more than 170 generations. All animal work has been conducted according to relevant national and international guidelines.

### RNA extraction and cDNA synthesis

Total RNA was extracted using the RNeasy Mini Kit (Qiagen, Valencia, CA) according to the manufacturer’s instructions. RNA concentration and purity were measured with the NanoDrop 1000 spectrophotometer (Thermo Fisher Scientific, Rockford, USA) and the integrity was checked by agarose gel electrophoresis. A quantity of 1 μg of the total RNA was reverse transcribed to cDNA by using ReverTra Ace qPCR RT kit (Toyobo Co. LTD, Osaka, Japan).

### Molecular cloning and sequence analysis

Based on *N*. *lugens* genome and transcriptome [35, 36], one RanGTPase homology (a single copy gene) was identified and the sequence was confirmed by reverse transcription polymerase chain reaction (RT-PCR) using primers listed in Table 1. The PCR product was gel purified, ligated into the vector TOPO2.1 (Invitrogen, Carlsbad, CA) and transformed into *Escherichia coli* DH5α competent cells (Novagen, Darmstadt, Germany). Ten recombinant plasmids from several independent subclones were fully sequenced on the Applied Biosystems 3730 automated sequencer (Applied Biosystems, Foster City, USA) from both directions. The resulting sequence (*NlRan*) was submitted to GenBank (KT313028).

**Table 1:** Primers used for RT-PCR, dsRNA synthesis and qRT-PCR.

Primer name	Forward sequence (5'-3')†	Reverse sequence (5'-3')†	Amplicon size (bp)
**RT-PCR**			
*NlRan*	CCATTGTGGACCGTAACGTCT	ACGCGTATTTTGCACTCGAAC	1426
**dsRNA synthesis**			
ds*NlRan*	T7-AGTATGTTGCCACCCTTGGAG	T7-ATTGTGACCTCGGGAGGGAG	454
ds*GFP*	T7-CCTGAAGTTCATCTGCACCAC	T7-TGATGCCGTTCTTCTGCTTGT	355
**qPCR**			
q*NlRan*	GAGAAGCCGTTCCTGTGGTT	AACAAGTTCGCACGGTTGATT	260
q*NlFTZ-F1*	GCTACCACTATGGCCTGCTC	TTGTCGTTGGCGCATCATTT	278
q*NlKr-h1*	CAAGTGTGGTGCCATAGGTC	CCTCCCTCCGTATTGAACAT	131
q*NlVg*	AGTCAACTACAAGCAGGGAGCAGTG	GCTCATCAACATCGTAGTGGGTCTC	235
q*NlRPS11*	CCGATCGTGTGGCGTTGAAGGG	ATGGCCGACATTCTTCCAGGTCC	159
q*NlRPS15*	TAAAAATGGCAGACGAAGAGCCCAA	TTCCACGGTTGAAACGTCTGCG	150

Note: T7 promoter sequences, 5**'**-TAATACGACTCACTATAGGGAGA-3**'**.

The theoretical isoelectric point and molecular weight of the deduced NlRan protein were calculated using ExPASy [37]. Homologues from *Drosophila melanogaster*, *Aedes aegypti*, *Apis mellifera*, *Harpegnathos saltator*, *Riptortus pedestris*, *Helicoverpa armigera*, *Bombyx mori*, *Acyrthosiphon pisum* and *Pediculus humanus corporis* were aligned with *NlRan* using ClustalW2 [38].

### dsRNA synthesis and bioassay

A 454 bp of *NlRan* cDNA and a 355 bp green fluorescent protein (*GFP*) fragments were amplified by PCR using specific primers conjugated with the T7 RNA polymerase promoter (5′-taatacgactcactataggg-3′) (primers listed in Table 1). This targeted region was further BLAST (BLASTN) searched against the *N*. *lugens* draft genome to identify any possible off-target sequences that had an identical match of 20 bp or more. The products were gel purified and used as templates to synthesis dsRNA, using MEGAscript T7 High Yield Transcription kit (Ambion, Austin, USA). The quality and concentration of dsRNA were determined by agarose gel electrophoresis and the Nanodrop 1000 spectrophotometer and kept at -70°C until use.

A previously reported procedure was used to carry out dsRNA injection *in vivo* bioassay [39]. Briefly, ds*NlRan* (stock solution estimated at 4.000 mg/mL), ds*GFP* (estimated at 4.000 mg/mL, negative control), or double distilled water (blank control) was injected into the thorax between the mesocoxa and the hind coxa of third-instar nymphs (2-day old), fourth-instar nymphs (2-day old) or fifth-instar nymphs (3-day old) in volumes of 0.025 μL, 0.05 μL and 0.1 μL, respectively, with different doses. One day after injection, dead individuals (assumed to be caused by physical damage) were discarded (for the current study, no mortality was found in treatment groups, and 2% mortality was found in control groups), and the survived ones were observed daily for survival rate, nymph development and phenotype formation. Furthermore, the weight gain and honeydew excretion in 48 h, ovary development and fecundity of the emerged female adults from dsRNA injected 5th instar nymphs were assessed. All treatments were performed with 10 replicates (25 nymphs per replication and a total of 250 nymphs per treatment). The experiment was repeated three times as independent biological replicates.

### Real-time quantitative PCR (qPCR)

Total RNA samples were prepared from eggs, each day of the first- through fifth-instar nymphs and newly emerged female adults (FeAd), and from integument (In), midgut (Mg), leg (Lg), fat body (Fb) of normal fifth-instar nymphs and adults, and ovary (Ov). Furthermore, total RNA was extracted from nymphs at 4 days after dsRNA injection. The mRNA levels of *NlRan*, hormone response genes, *FTZ-F1*, *NlKr-h1* and *vitellogenin* (*NlVg*) were estimated by qPCR, using ribosomal protein S15e (*rps15*) and *rps11* as internal control genes [40]. All qRT-PCR primers were listed in Table 1. No template was added to negative control reactions. Each sample was repeated in biological and technical triplicates and the transcriptional levels of those genes were calculated by the 2^−ΔΔCT^ method [41].

### Data analysis

Data were analyzed using the Data Processing System software [42]. The student’s *t*-test was applied for comparing two samples, and one-way analysis of variance (ANOVA) with protected Fisher’s least significant difference (LSD) test was applied when more than two samples were compared.

## Results

### Identification of the *Ran* gene of *N*. *lugens*

**Fig 1:**
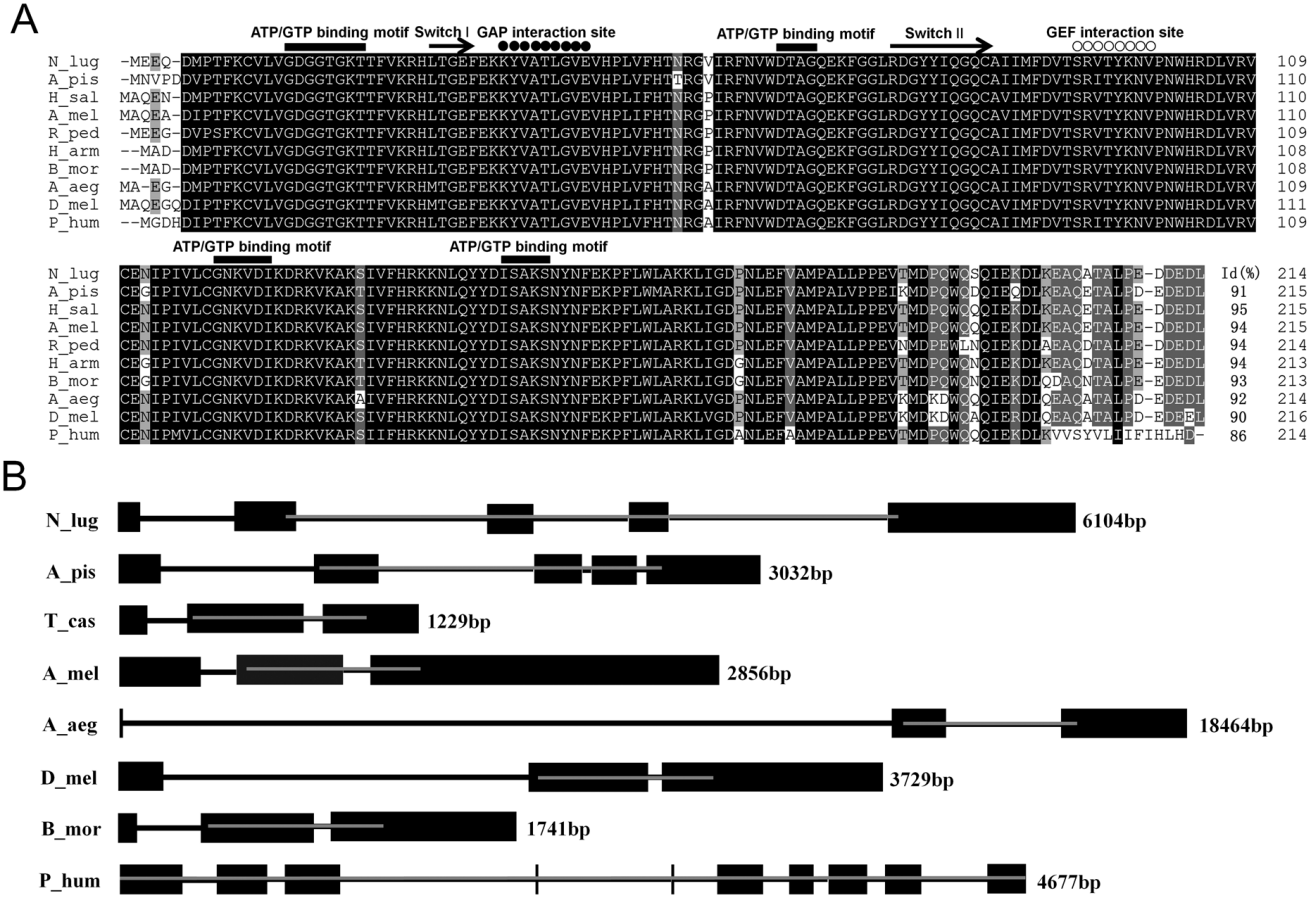
Sequence alignment and gene structure comparison of *Ran*. A: Rans from *N*. *lugens* (*N_lug*), *Drosophila melanogaster* (*D_mel*, AAF30287.1), *Aedes aegypti* (*A_aeg*, XP_001659895.1), *Apis mellifera* (*A_mel*, XP_393761.1), *Harpegnathos saltator* (*H_sal*, EFN84506.1), *Riptortus pedestris* (*R_ped*, BAN20580.1), *Helicoverpa armigera* (*H_arm*, ACN67046.1), *Bombyx mori* (*B_mor*, NP_001040274.1), *Acyrthosiphon pisum* (*A_pis*, NP_001155556.1) and *Pediculus humanus corporis* (*P_hum*, XP_002423913.1). The domain elements are marked with filled rectangles. Amino acids with 100%, >80%, and >60% conservation are shaded in black, dark gray and light gray, respectively. Gaps have been introduced to permit alignment. B: Exons and introns are denoted with black boxes and lines, respectively, the protein-coding regions are indicated with gray lines. The genome sequence data were downloaded from GenBank with the follow accession numbers: KN153157 (*N*. *lugens*), ABLF02039222 (*Acyrthosiphon pisum*), AAJJ01002638 (*Tribolium castaneum*), AADG06001923 (*Apis mellifera*), CH477572 (*Aedes aegypti*), AE014298 (*Drosophila melanogaster*), BABH01008630 (*Bombyx mori*) and DS235063 (*Pediculus humanus corporis*).

A cDNA of putative *Ran* gene in *N*. *lugens* was cloned (*NlRan*). The *NlRan* contains a complete coding region and encodes 214 amino acid residues. The deduced protein was predicted with molecular weight and isoelectric point of 24.52 kDa and 6.96, respectively. NlRan contains four ATP/GTP binding motifs, two effector molecular binding motifs (GAP and GEF interaction site) and two switch regions (Switch I and II). Two interaction sites, GTPase-activating protein (GAP) and guanine-nucleotide-exchange factor (GEF) sites are located at position 36–44 and 92–99, respectively, which are conserved among various insect Rans (Fig 1A). The highly acidic C-terminal motif EDDEDL can be found at positions 209–214, though it was slightly modified from DEDDDL that exited in other insects.

The multiple sequence alignment showed that NlRan shares the highest identity with that of *Harpegnathos saltator* (95.8%), followed by 95.3–87.3% identities with that of *D*. *melanogaster*, *A*. *aegypti*, *A*. *mellifera*, *R*. *pedestris*, *H*. *armigera*, *B*. *mori*, *A*. *pisum* and *P*. *humanus corporis* (Fig 1A).

Analysis of the genomic position and structure showed that *NlRan* is located between 119 kb to 125 kb on scaffold1292 (GenBank accession no. KN153157) [36]. *NlRan* possesses five exons and four introns, which are similar with that from *A*. *pisum* (Fig 1B). One less exons or introns of *Ran*, however, were observed from *T*. *castaneum*, *A*. *mellifera*, *A*. *aegypti*, *D*. *melanogaster* and *B*. *mori*. Eight exons and seven introns are present in that of *P*. *corporis*, which is shorter than *NlRan* in length.

### Temporal and spatial expression profiles

**Fig 2:**
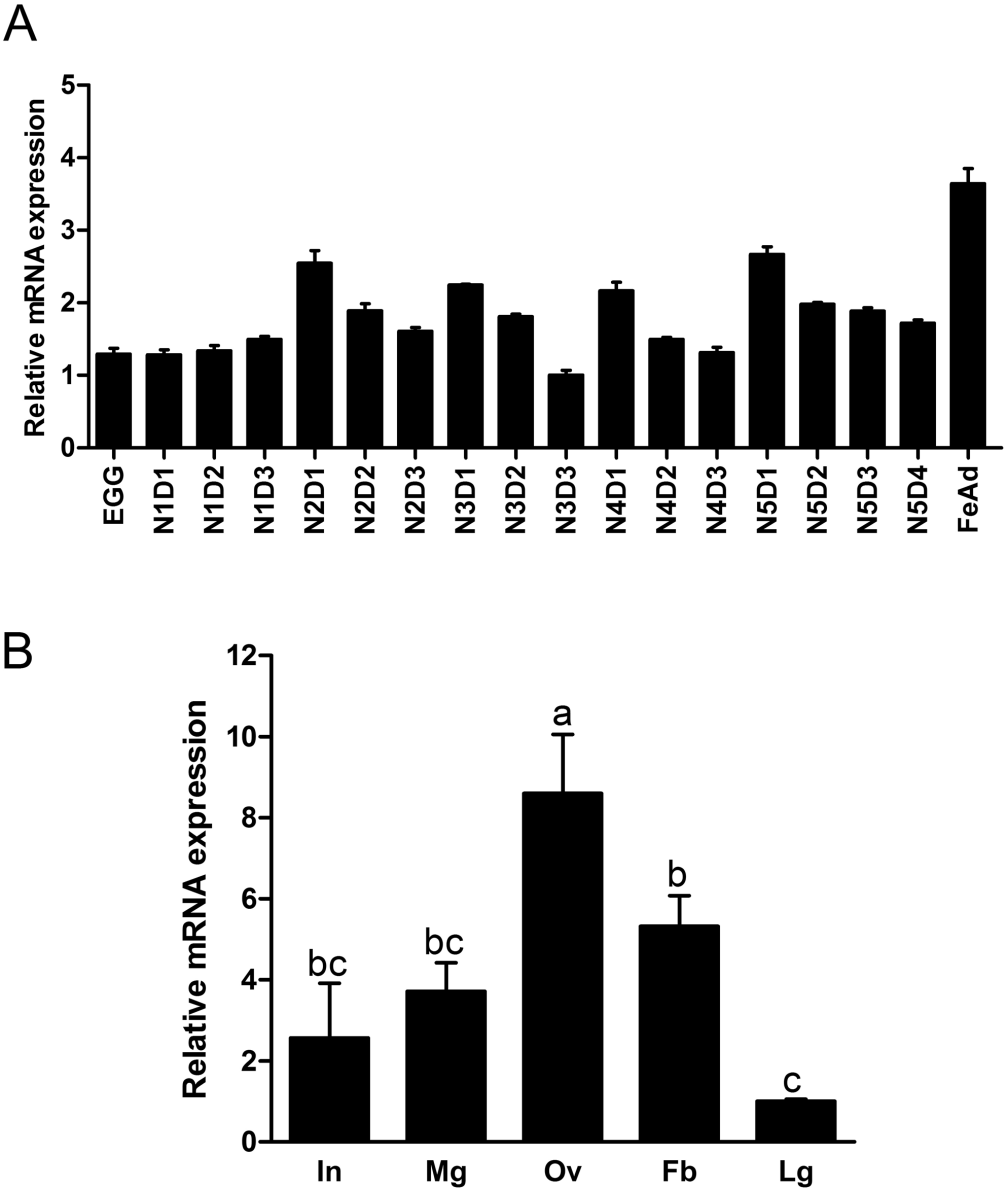
Temporal and spatial expression patterns of *NlRan*. A) Relative expression level of *NlRan* at different developmental stages. N1D1 (day one of first-instar nymph) to N5D4 (day 4 of fifty-instar nymph) refers to first- to fifth-instar nymphs of different age, FeAd, newly emerged female adult. B) Relative expression levels of integument (In), midgut (Mg), ovary (Ov), leg (Lg), fat body (Fb) of 2-day old female adults. The bars represent 2^−ΔΔCT^ values (±SE) normalized to the geometrical mean of housekeeping gene expression. SE was determined from 3 independent biological replicates, each with three technical replications. Different letters indicate a significant difference at P value <0.05.

The expression levels of *NlRan* were examined throughout the developmental stages, including eggs, the first- to fifth-instar nymphs and newly emerged female adults. *NlRan* was ubiquitously but unevenly expressed in nymphs and adults. Started at 2nd instar, the expression levels of *NlRan* was higher in the newly-molted individuals and decreased gradually as they grew until the next molt. The highest level was found in newly emerged female adults (Fig 2A). *NlRan* expressed the highest level in ovary, followed by fat body, midgut and integument, and the lowest level in legs (Fig 2B) (P<0.05, ANOVA protected Fisher’s LSD test).

### Effect of dsRNA on the expression of *NlRan* and downstream genes

**Fig 3:**
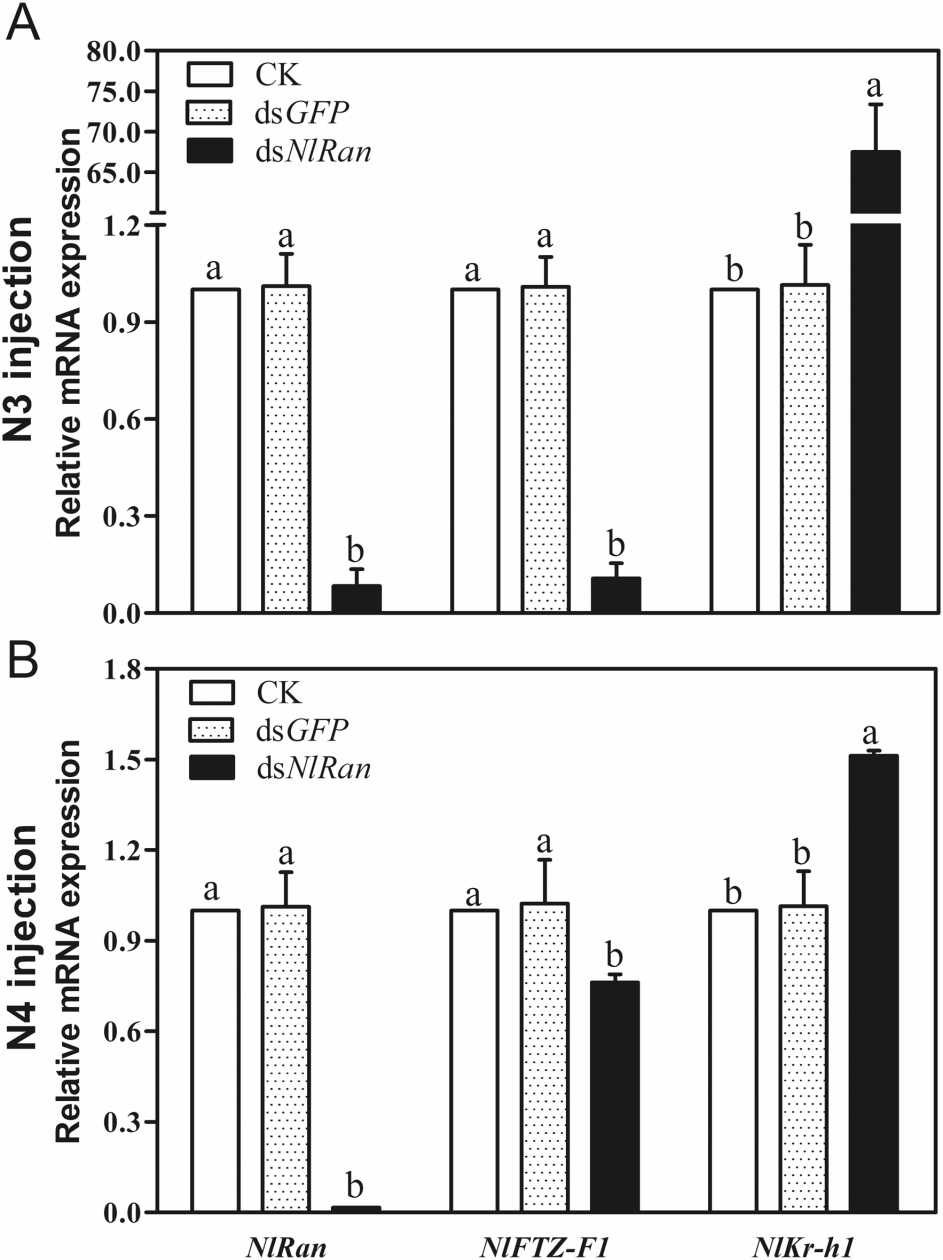
Effect of ds*NlRan* on the expression levels of *NlRan*, *NlFTZ-F1*, *NlKr-h1* in injected third-instar (N3) (A) and fouth-instar (N4) nymphs (B) at four days after injection. The expression levels of 5–10 individuals were determined by qPCR. The bars represent 2^−ΔΔCT^ values (±SE) normalized to the geometrical mean of housekeeping gene expression. SE was determined from 3 independent biological replicates, each with three technical replications. Different letters indicate a significant difference at P value <0.05.

After four days of ds*NlRan* injection, *Ran* transcript levels in the surviving nymphs were significantly reduced. In the third-instar nymphs, injection of ds*NlRan* (0.025 μL) at the concentrations of 0.001, 0.010, 0.100 and 1.000 mg/mL suppressed *NlRan* expression level by 85.7%, 74.1%, 94.3% and 91.7%, respectively, comparing with the blank control. In contrast, *NlRan* expression levels in ds*GFP* injected planthoppers remained unchanged (S1 Fig). Concentration of 0.100 mg/mL was selected to inject the fourth-instar nymphs. The mRNA level of *NlRan* in the treated nymphs were significantly reduced by 98.4% (Fig 3B), comparing with the blank control. Similarly, the *NlRan* expression level varied little in ds*GFP*-injected nymphs (P<0.05, ANOVA protected Fisher’s LSD test).

As expected, *NlFTZ-F1* expression levels in nymphs treated at the third- and fourth instar stage were reduced by 89.3% and 23.8%, respectively. The former is significantly silenced, comparing to the blank control. In the meanwhile, *NlKr-h1* mRNA levels were significantly up-regulated by 67.0 folds in the third instar nymphs and 1.5 folds in the fourth instar nymphs (Fig 3A and 3B).

### Effect of ds*NlRan* injection on nymph survival and development

**Fig 4:**
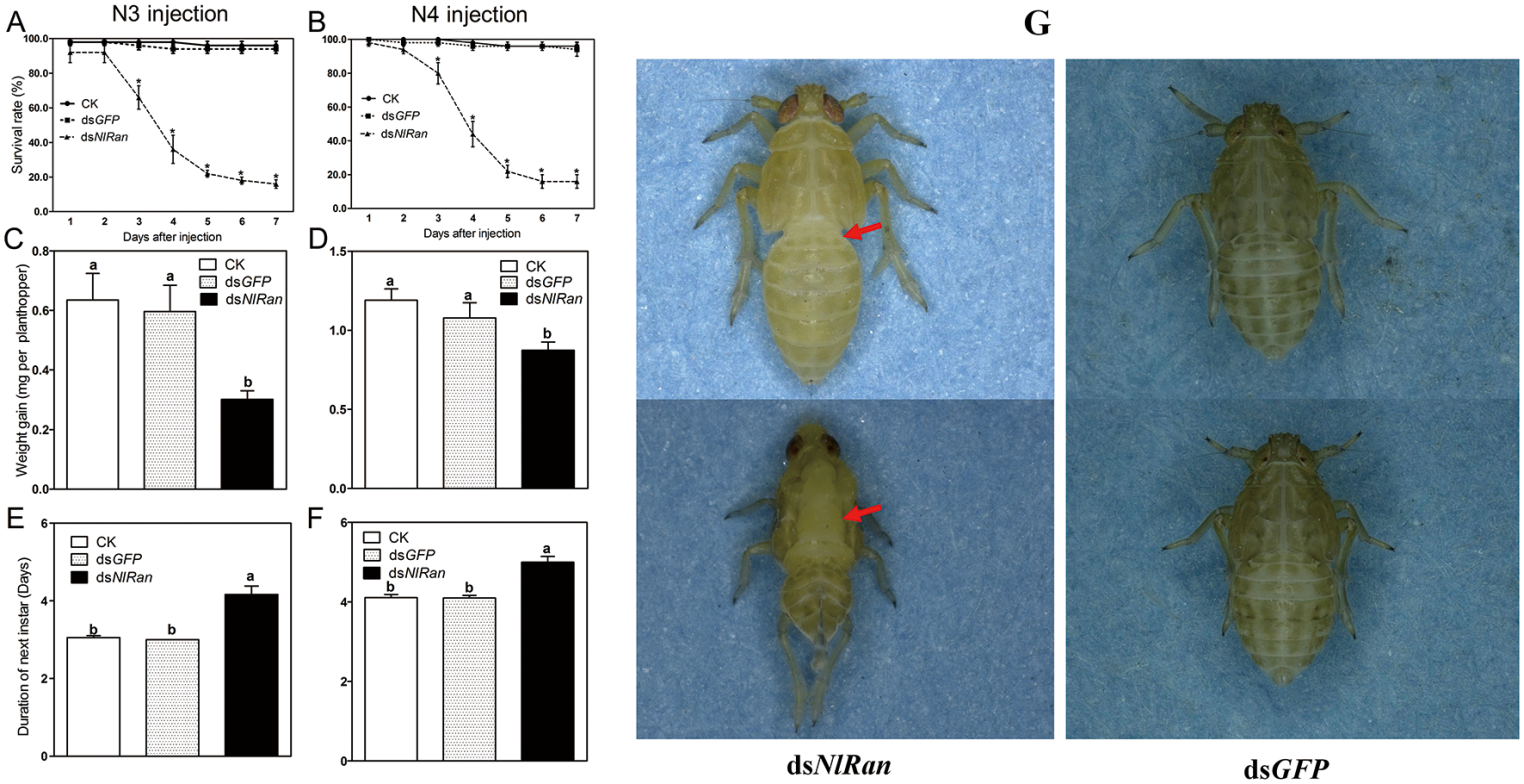
The performance of *N*. *lugens* nymphs subjected to ds*NlRan* injection. A, B: The survival rate (%) in N3 injection and N4 injection. C, D: Weight gain in 4 days (mg per individual) after N3 injection and N4 injection. E, F: Development duration (days) to the next molt (e.g. days between molts) after N3 injection and N4 injection, respectively. G: Two apparent phenotypic defects: the extended body forms and the old cuticle at notum split open but not complete shed off. The columns represent averages and SE. Different letters indicate a significant difference at P value <0.05.

The survival and development of the ds*NlRan* injected third- and fourth-instar nymphs were examined. ANOVA analysis revealed significant effects at three days after injection and beyond. The survival rates at the seventh day were only 14.0% and 12.0% for the third- and fourth-instar nymphs, respectively (Fig 4A and 4B) (P = 0.0019; P = 0.0265). All individuals were died at the tenth day.

The development of nymphs injected with ds*NlRan* was delayed significantly. The weight gain of nymphs treated at the third- and fourth-instar stage were significant reduced by 50% and 26%, respectively, compared with the blank control, while ds*GFP* injected nymphs were similar compared to the blank control (Fig 4C and 4D) (P = 0.0008; P = 0.0082). The duration of the fourth- or fifth-instar nymphs of *NlRani* treated at third- and fourth-instar stage were 4.2 and 5.0 days, respectively, statistically longer than the blank control (3.1 and 4.1 days, respectively) (P = 0.0000) (Fig 4E and 4F).

Three days after injection, all individuals in the blank and ds*GFP* controls were successfully molted to the next stage and showed normal morphology. In contrast, on average 57.8% of ds*NlRan*-treated nymphs failed to molt on time, and exhibited two apparent phenotypic defects: (1) Extended body form with a pitched first and perhaps second abdominal segments, and failed to shed their old cuticle and died; (2) The old cuticle at the notum split normally, but could not shed off, and evenly died (Fig 4G).

### Effect of ds*NlRan* injection on oogenesis

**Fig 5:**
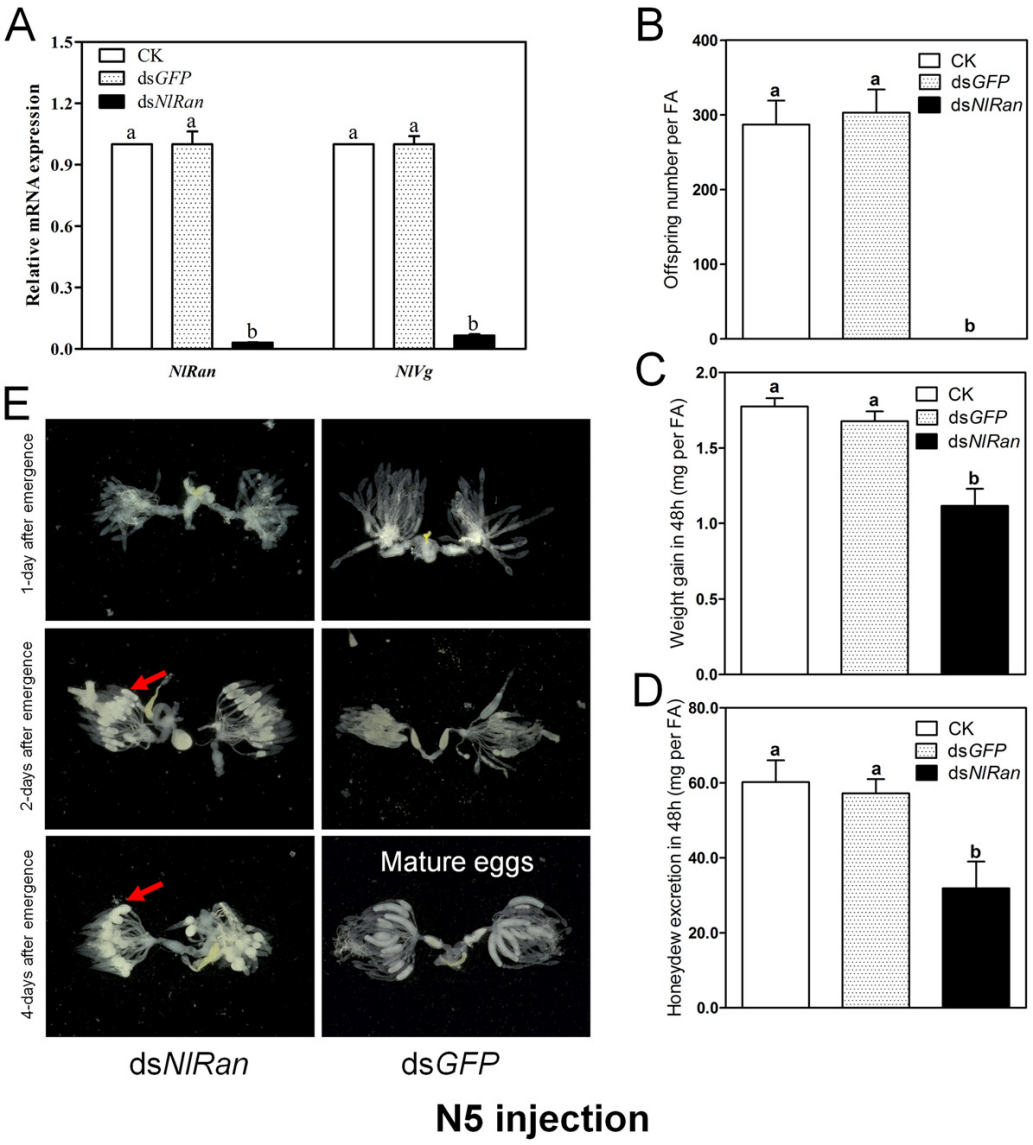
Effect of ds*NlRan* on the expression levels of *NlRan*, *NlVg* and *N*. *lugens* female performance and ovary development. A: The expression levels of ***NlRan and NlVg*** four days after dsRNA injection. B: Spawning numbers in N5 injection. C: Weight gain in 48 h (mg per individual) in N5 injection. D: Honeydew excretion in 48h (mg per female adult) in N5 injection. E: Abnormal ovary in ds*NlRan*-treated females.

Four days after ds*NlRan* injection, the mRNA level of *NlRan* in the treated fifth-instar nymphs were significantly reduced by 97.0%, comparing to the blank control. Since the *NlVg* mRNA is only expressed in female adults and nymphs treated with ds*NlRan* at the third- and fourth-instar stages cannot survive into adults, the expression level of *NlVg* was measured only for the fifty-instar treated adult survivals, and the result showed a significant decrease by 93.4% (Fig 5A). All the fifth-instar nymphs treated with ds*NlRan*, dsGFP or water successfully molted into adults. The fecundity, weight gain and honeydew excretion of these adults were examined. The results revealed that ds*NlRan*-treated females had no offspring (no eggs or nymphs observed), while the adults of the blank control and negative control had similar number of offspring, 278 and 303, respectively (Fig 5B). The ds*NlRan* treatment significantly reduced the weight gain and honeydew excretion of the females by 1.16 mg and 31.87 mg, respectively, comparing to the blank control (Fig 5C and 5D).

Dissection of the females treated with ds*NlRan* at the fifth-instar stage revealed that the ovary development of all individuals was abnormal: the oocytes remained at primitive stage with no eggshells (Fig 5E).

## Discussion

In the present paper, we cloned and characterized *Ran* gene in *N*. *lugens*. The Ran is important for all organisms as it is essential for nuclear assembly, nuclear transport, spindle assemble, and mitotic regulation. Interestingly, it has been shown to participate in the 20E signal transduction pathway by regulating the location of EcR-B1 in *Helicoverpa armigera* [14]. So Ran may participant in theecdysteroid-signaling pathway, which plays a critical role in insect development and reproduction. The RNAi based gene knockdown of the current study showed that *NlRan* can be successfully silenced through ds*NlRan* injection. Using this approach we provided, for the first time, direct evidence to support the hypothesis that NlRan is involved in the development and reproduction of *N*. *lugens*. Firstly, NlRan has the typical protein motifs, active sites and gene structure of insect *Ran*. *NlRan* has four ATP/GTP binding motifs, two effector molecular binding motifs (GAP and GEF interaction site) and two switch regions (Swith I and II), similar with the structure of Ran orthologs [11, 43]. ATP/GTP binding motif is the signature of phosphate-binding loop, and GAP and GEF interaction site is effector binding site, which are common in GTPase superfamily [3, 4, 44]. The switch I and II regions have been defined as those regions that change their structure between the GTP- and GDP-bound conformation [45]. These structural features indicate that NlRan can switch its activity by RanGDP and RanGTP transformation. Moreover, the temporal and spatial expression patterns suggest an association with insect development and reproduction. Throughout the development of *N*. *lugens*, the Ran gene was expressed universally with higher levels at the beginning of each nymphal molt and in female adults. Among the tissues examined, *NlRan* also was expressed universally with the highest level in ovaries and the lowest level in legs. This expression profile of *NlRan* gene is similar to those of 20E signal transduction pathway and JH biosynthesis in Hemimetabolan species [28, 46].

Secondly, we found that ds*NlRan* knocked out the targeting gene and its down-stream genes. Compared with the blank control, the mRNA level of nymphs treated with ds*NlRan* injection at the third- and fourth-instar stages was dramatically decreased. The ds*NlRan* also significantly inhibited the mRNA level of *NlFTZ-F1* and increased the mRNA levels of a down-stream gene *Kr-h1* that is involved in JH cascade. In *H*. *armigera*, knockdown of *Ha-Ran* resulted in the suppression of 20E regulated genes including *EcR* [43]. The levels of *EcR* from insects that were treated with ds*EcR* concomitantly decreased with the levels of *FTZ-F1* transcripts [47]. The decreased levels of *FTZ-F1* lowered 20E titer, and induced the expression of a JH biosynthesis gene, hence increased JH titer [32]. Furthermore, *Kr-h1* gene expression was regulated through JH-Met-Kr-h1 pathway, which mediates metamorphic transformations [33, 48, 49]. Our results support the hypothesis that *NlRan* was involved in 20E signal transudation pathway, and was cross talking with JH pathway to control hormone balance during development and reproduction.

At *in vivo* level, the knockdown of *NlRan* in the third and fourth instar nymphs caused significant phenotypic impairment of *N*. *lugens* with two apparent defects in body formation and molting process that were fatal. Similar phenotypes have been documented in insects including *Blattella germanica* and *L*. *decemlineata*, after knockdown of *FTZ-F1*, a 20E signal transduction pathway gene [32, 50]. This further suggests that Ran is an important factor in the regulation of hormonal balance that is essential to the normal physiology of insects.

Thirdly, the fifth instar nymphs that were treated with *dsNlRan* had no offspring at adult stage. The dissection revealed significantly reduced ovariole development and vitellogenin production with no eggshell. These observations corresponded well with that *NlRan* transcript level knockdown significantly inhibited the mRNA level of *NlVg* because the biosynthesis of *Vg* is important for egg laying insects as their reproductive capacity depends heavily on *Vg* accumulation in the oocytes [51–54]. Apparently, factors other than Ran may also cause abnormal ovarioles, which we plan to study in the near future.

RNAi-based knockdown of functional genes by injection of dsRNA have been successful in *N*. *lugens* [26–30, 36]. This current study added Ran (up to 0.025 ng of the minimum volume, seen in the S1 Fig) in the examined gene list that includes *calreticulin*, *cathepsin-B*, *nicotinic acetylcholine receptors β2 subunit*, *chitin synthase*, *EcR*, *CYP6AY1*, *flightin*, *glutathione S-transferase*, *NADPH-cytochrome P450 reductase*, *chitin deacetylases*, *Vg* and *Vg receptor*, *insulin-like* and *insulin-like receptor*, *forkhead box*, *phosphatase and tensin homolog deleted on chromosome ten*, *chico*, *AKT*, *LnK*, *Tor*, *Raptor*, *Raf*, *ErK* and *Bicaudal-C* with the volume of 0.05–250 ng dsRNA [26–30, 34, 36, 55–58]. To our limited knowledge, Ran requires a low volume of dsRNA to efficiently knock down the target gene, which is only next to *Bicaudal-C* gene. The silencing of Ran during nymphal development was fatal and stopped reproduction completely. Compared with other studied genes, Ran seems a better candidate as a target for pest control purpose if a level of selectivity (targets vs. non targets) can be installed.

In summary, we identified a putative Ran and revealed its potential roles in the development and reproduction of *N*. *lugens*. In addition to *EcR* gene in 20E signal transduction pathway [24], *NlRan* maybe a potential target for RNAi-based pest control. More relevant research is in progress.

## Supporting Information

S1 Fig The survival rates (A) and *NlRan* mRNA abundance (B) of *N*. *lugens* that treated with different concentrations of ds*NlRan* at the third-instar nymph stage. Total RNAs were isolated from 5–10 nymphs subjected to ds*NlRan*, dsGFP and double distilled water injection. The bars represent 2^−ΔΔCT^ values (±SE) normalized to the geometrical mean of housekeeping gene expression. SE was determined from 3 independent biological replicates, each with three technical replications. Asterisks indicate significant difference between values at ** = 0.01 or * = 0.05 of P values. Different letters indicate a significant difference at P value <0.05.
(TIF)
