# Supplementary figures and images for: Ran Involved in the Development and Reproduction Is a Potential Target for RNA-Interference-Based Pest Management in Nilaparvata lugens
Source: PLoS One. 2015 Nov 10;10(11):e0142142. doi: 10.1371/journal.pone.0142142 (PMC4640576; doi:10.1371/journal.pone.0142142)

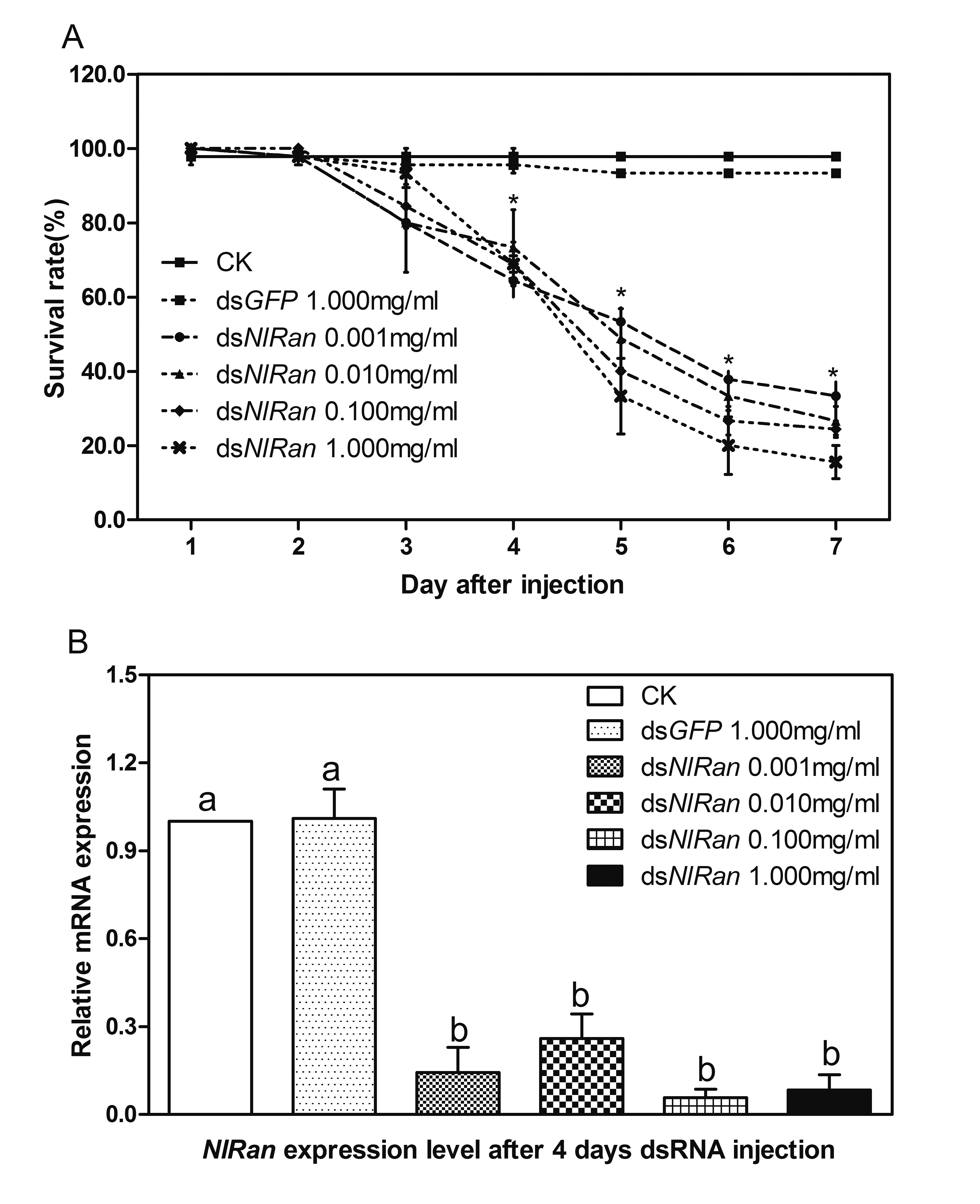

Supplement: S1 Fig — Total RNAs were isolated from 5–10 nymphs subjected to dsNlRan, dsGFP and double distilled water injection. The bars represent 2−ΔΔCT values (±SE) normalized to the geometrical mean of housekeeping gene expression. SE was determined from 3 independent biological replicates, each with three technical replications. Asterisks indicate significant difference between values at ** = 0.01 or * = 0.05 of P values. Different letters indicate a significant difference at P value <0.05. (TIF) [file pone.0142142.s001.tif]
